# Multicentric Castleman’s disease in human immunodeficiency virus infection: two case reports

**DOI:** 10.1186/s13256-018-1656-5

**Published:** 2018-05-05

**Authors:** Amanda Caroline Ribeiro Sales, Valter Romão de Souza Junior, Marta Iglis de Oliveira, Claudia Azevedo Braga Albuquerque, Evônio de Barros Campelo Júnior, Paulo Sérgio Ramos de Araújo

**Affiliations:** 1 Federal University of Pernambuco, Av. Prof. Moraes Rego 1235, Recife, 50670-901 Brazil; 2 University of Glasgow, University Avenue, Glasgow, G12 8QQ UK; 3 Universidade Federal De Pernambuco, Rua Capitão Aurélio de Araújo 325, AP 202 BL D, Recife, Pernambuco 50731230 Brazil

**Keywords:** Castleman’s disease, HIV, Lymph nodes enlargement, HHV-8

## Abstract

**Background:**

Castleman’s Disease is a rare B-cell lymphoproliferative disease. It is mostly benign and is characterized by non-neoplastic lymph node hypertrophy, associated with infection by human herpesvirus-8 in people with the human immunodeficiency virus/acquired immunodeficiency syndrome. Although the unicentric or localized form presents as benign, the multifocal form can manifest severe systemic symptoms. We report two unusual cases of men presenting cervical enlarged lymph nodes that were believed to be infectious.

**Case presentation:**

The first case is a 41-year-old feoderm man who presented to the Department of Infectious Diseases of the Hospital das Clínicas in May 2015, with irregular fever history (38–39 °C), dyspnea, weight loss (8 kg/1 year), and asthenia with increased cervical lymph nodes of 1-year duration. His immunohistochemical diagnosis presented Castleman’s disease in plasmacytic/diffuse form. In the second case, a 35-year-old feoderm man presented at the same hospital with multiple cervical enlarged lymph nodes and histopathological evidence of Castleman’s disease associated with human herpesvirus-8.

**Conclusion:**

Considering the importance of differential diagnosis of lymphoid disorders, Castleman’s disease is a challenging diagnosis in people living with human immunodeficiency virus/acquired immunodeficiency syndrome and can be easily misdiagnosed when lymphoid disorders are present in the human immunodeficiency virus/acquired immunodeficiency syndrome population due to nonspecific symptoms and signs.

## Background

Castleman’s disease (CD) was described in 1954 in a case report of a 40-year-old man presenting a mediastinal mass characterized histologically as lymph node hyperplasia and follicles with small hyalinized foci [1, 2]. In the United States of America (USA), the incidence of CD is estimated to be approximately 21 to 25 cases/million people per year, with 23% of these cases potentially representing the multicentric form of CD [3, 4]. CD is classified into unicentric and multicentric forms. The unicentric or localized form, the most common type, occurs around 35 years of age [5], involving only one chain of lymph nodes. Thoracic, mediastinal, or abdominal lymph nodes are mostly affected. In the multicentric or disseminated form, the most affected age group comprises individuals between 50 and 60 years of age [6], in which there is involvement of a larger number of lymph nodes with more important systemic involvement. CD can result in malignant neoplasms such as Kaposi’s sarcoma (KS), non-Hodgkin’s lymphoma, Hodgkin’s lymphoma, and polyneuropathy, organomegaly, endocrinopathy, monoclonal gammopathy, and skin changes (POEMS) syndrome. There are three histological variants: hyaline vascular, plasma cells, and mixed [7, 8]. The literature data about multicentric CD (MCD) are limited for patients in their fourth and fifth decades of life. We present two rare cases of MCD in a 41-year-old feoderm men and a 35-year-old feoderm men, which were initially thought to be due to tuberculosis.

## Case presentation

### Case 1

A 41-year-old feoderm man diagnosed with human immunodeficiency virus (HIV)/acquired immunodeficiency syndrome (AIDS) since 2000 and on antiretroviral therapy (ART) with tenofovir, lamivudine, and lopinavir/ritonavir presented to the Department of Infectious Diseases of the Hospital das Clínicas in May 2015, with irregular fever history (38 to 39 °C), dyspnea, weight loss (8 kg/1 year), asthenia, and enlarged and painless cervical lymph nodes of 1-year duration. Rifampicin, isoniazid, pyrazinamide, and ethambutol were prescribed for empiric treatment of ganglionic tuberculosis in the last 6 months but with no clinical response. Six months after the end of treatment, he developed hemolytic anemia and papular violaceous skin lesions in his upper limbs where a biopsy revealed KS.

Thorax computed tomography (CT) showed enlargement of axillary and mediastinal lymph nodes. An abdomen CT scan presented intracavitary fluid with extensive hypertrophied retroperitoneal lymph nodes, either mesenteric root or inguinal. There was also liver and spleen enlargement with a splenic index of 590. A cervical lymph node biopsy revealed by immunohistochemistry (IHC) a CD diagnosis, diffuse plasmacytic form. IHC did not show malignant lymphoproliferative disease and marked the presence of human herpesvirus-8 (HHV-8) predominantly in mantle zone cells.

He was submitted to a chemotherapy protocol with four cycles of rituximab 700 mg/cycle, one dose weekly, associated with ganciclovir 5 mg/kg twice daily during the first week, and then 5 mg/kg per day for another 3 weeks. He presented a favorable clinical response, evolving with improvement of fever, and reduction of spleen and liver.

### Case 2

A 35-year-old feoderm man diagnosed with HIV/AIDS since 2011 on ART with abacavir, lamivudine, and efavirenz presented to the Department of Infectious Diseases in the Hospital das Clínicas with fever, asthenia, weight loss, and progressive enlargement of anterior cervical lymph nodes and axillary regions, measuring 7 cm to the right and 5 cm to the left, painless to palpation. He was prescribed rifampicin, isoniazid, pyrazinamide, and ethambutol for empiric treatment of ganglionic tuberculosis and at the end of 2 months it persisted without clinical improvement and with persistence of fever. An abdominal ultrasound showed enlarged lymph nodes and increased spleen which contained multiple hypoechoic nodules. Cervical ultrasound visualized multiple enlarged lymph nodes in all the chains. A chest and abdomen CT scan showed enlarged lymph nodes in his armpits, in his mediastinum, and retrocrural space, besides splenomegaly. Incisional biopsy of the lymph node showed marked angiofollicular hyperplasia and plasmacytosis compatible with CD, a plasma cell variant.

He was submitted to a chemotherapy protocol with six cycles of 21 days with cyclophosphamide, doxorubicin, vincristine, and prednisone (CHOP). In the follow-up, chest, abdomen and neck CT scans were normal and he had a satisfactory clinical response with disease in remission.

## Discussion

The majority of patients diagnosed with Castleman's Disease (CD) are typically between 30 and 40 years (middle-aged individuals). The etiopathogenesis is poorly understood although there can be association with HHV-8 and HIV infection [1]. One of the explanations is a reactive hyperplasic lymphoid initiated by chronic antigenic stimulation in association with a viral infection mainly in the respiratory and gastrointestinal tract. Another hypothesis suggested that the cause might be a lack of immune regulation with increased expression of the interleukin (IL)-6 gene, a cytokine with pleiotropic effects on the immunological system and hematopoiesis, which is related to the etiology of multiple myeloma [2].

Regarding the histological classification, there are three variants: hyaline vascular, plasma cell, and mixed variant. Hyaline vascular are multiple tight aggregates of follicular dendritic cells with concentric form. Plasma cell variants show hyaline vascular changes with polytypic plasma cells. The histological plasma form, found in both cases in this study, occurs around 10% in the unicentric form and in 80–90% in the multicentric form [1]. Regarding the age group, it can affect individuals of any age; the localized form of the disease has a higher incidence in adolescents and young adults, whereas the multicentric form affects older individuals and patients with immunodeficiency, especially in AIDS, more, as observed in both cases of this report. Individuals living with HIV/AIDS appear to be at increased risk of developing multicenter CD, which usually arises concomitantly with KS as occurred in the first case reported in this study [3].

When present in the multicentric form, it is usually symptomatic, due to the release of cytokines: tumor necrosis factor (TNF)-alpha, IL-1, and IL-6. In both cases, asthenia, weight loss, and fever were recorded as the main clinical manifestations of CD [3, 4]. Polyadenopathy is common and presents on average with four involved sites and is frequently associated with hepatosplenomegaly [3, 4]. Laboratory test results usually indicate thrombocytopenia, hypergammaglobulinemia, hypoalbuminemia, and anemia. Diagnosis can only be defined by histological examination of the ganglion or tissue affected by the lymphoproliferative process. Some thymomas and lymphomas have a similar appearance to CD in anatomopathological examination, and an immunohistochemical study is sometimes necessary to confirm the diagnosis.

From a clinical and imaging perspective, CD is indistinguishable from other lymphoproliferative diseases. It also presents radiological and surgical features similar to: other diseases including neoplasms, reactive lymph node hyperplasia, and HIV infection; autoimmune diseases, such as rheumatoid arthritis and Sjögren’s syndrome; inflammatory diseases, such as sarcoidosis and tuberculosis; or neoplasms such as neurofibroma; and Hodgkin’s lymphoma or non-Hodgkin’s lymphoma. Syndromic symptoms misdiagnosed as tuberculosis have CD as an important differential diagnosis, which is often described in the literature [3].

Localized CD is treated by surgical excision that allows complete recovery without relapse in almost all cases [3]. However, there is no therapeutic consensus for MCD and several treatments such as surgery, corticosteroid therapy, and chemotherapy are proposed, often in combination [3]. Anti-IL-6 antibody has also been successfully tested for the relief of systemic manifestations in patients who are HIV/HHV-8 negative [3, 6, 7].

Ganciclovir, interferon-α, or rituximab may be the best option for patients with MCD with HHV-8 infection [8], while CHOP or cyclophosphamide, vincristine, doxorubicin, and dexamethasone (CVAD) may be better suited for patients with severe systemic manifestations [9]. Considering the limited experience in the treatment of CD, the administration of anti-CD20 monoclonal antibody (rituximab) was chosen in the first case presented, due to the impossibility of surgical resection of the mass, as this option is pointed out in the literature as effective in the treatment of immune manifestations, such as hemolytic anemia associated with CD [8]. Hemolytic anemia may possibly be a consequence of the production of cytokines that inhibit hematopoiesis. In the second case, it was decided to use the combination of CHOP, since some authors suggested starting treatment with corticosteroids and chemotherapy [9–11].

Unlike the localized form, the plasma type has a less favorable prognosis and its clinical evolution is frequently aggressive and fatal due to the development of infections and neoplasias, such as KS and lymphomas [3, 9, 12]. Our two patients had a satisfactory evolution and to date there has been no relapse of the disease.

## Conclusions

The reports of these cases reinforce the need to include CD in the differential diagnosis of lymphoproliferative diseases in individuals living with HIV/AIDS, requiring a high degree of clinical suspicion in order to arrive at the correct diagnosis and management. MCD may have a poor prognosis and depend on its stage at the moment of diagnosis, reinforcing the importance of histological diagnosis in patients presenting with lymphadenopathy.

## Abbreviations

AIDS: Acquired immunodeficiency syndrome
ART: Antiretroviral therapy
CD: Castleman’s disease
CHOP: Cyclophosphamide, doxorubicin, vincristine, and prednisone
CT: Computed tomography
CVAD: Cyclophosphamide, vincristine, doxorubicin, and dexamethasone
HHV-8: Human herpesvirus- 8
HIV: Human immunodeficiency virus
IHC: Immunohistochemistry
IL: Interleukin
KS: Kaposi’s sarcoma
MCD: Multicentric Castleman’s disease
POEMS: Polyneuropathy, organomegaly, endocrinopathy, monoclonal gammopathy, and skin changes
TNF: Tumor necrosis factor
